# The association between disrespect and abuse of women during childbirth and postpartum depression: Findings from the 2015 Pelotas birth cohort study

**DOI:** 10.1016/j.jad.2019.06.016

**Published:** 2019-09-01

**Authors:** Mariangela Freitas Silveira, Marilia Arndt Mesenburg, Andrea Damaso Bertoldi, Christian Loret De Mola, Diego Garcia Bassani, Marlos Rodrigues Domingues, Alan Stein, Carolina V N Coll

**Affiliations:** a Postgraduate Program in Epidemiology, Federal University of Pelotas, Rua Marechal Deodoro, n° 1160, 3° andar, Pelotas CEP 96020-220, Brazil; b International Center for Equity in Health, Federal University of Pelotas, Brazil; c Postgraduate Program in Public Health, Federal University of Rio Grande, Brazil; d Centre for Global Child Health, Department of Pediatrics, The Hospital for Sick Children, University of Toronto, 555 University Ave, Toronto ON M5G 1×8, Canada; e Postgraduate Program in Physical Education, Federal University of Pelotas, Rua Luiz de Camões, n° 625, Pelotas CEP 96055-630, Brazil; f Department of Psychiatry, University of Oxford, Warneford Hospital, Warneford Ln, Oxford OX3 7JX, UK

**Keywords:** Disrespect and abuse, Childbirth, Postpartum depression, Institutional violence, Gender violence

## Abstract

**Background:**

This study examined the association between disrespect and abuse of women during facility-based childbirth and postpartum depression (PD) occurrence.

**Methods:**

We used data from the 2015 Pelotas (Brazil) Birth Cohort, a population-based cohort of all live births in the city. We assessed 3065 mothers at pregnancy and 3-months after birth. Self-reported disrespect and abuse experiences included physical abuse, verbal abuse, denial of care, and undesired procedures. We estimate the occurrence of each disrespect and abuse type, one or more types and disrespect and abuse score. The Edinburgh Postnatal Depression Scale (EPDS) was used to assess PD. EPDS scores ≥13 and ≥15 indicated at least moderate PD and marked/severe. Odds ratios (OR) were calculated by logistic regression.

**Results:**

The prevalence of at least moderate PD and marked/severe PD was 9.4% and 5.7%, respectively. 18% of the women experienced at least one type of disrespect and abuse. Verbal abuse increased the odds of having at least moderate PD (OR = 1.58; 95%CI 1.06–2.33) and marked/severe PD (OR = 1.69; 95%CI 1.06–2.70) and the effect among women who did not experience antenatal depressive symptoms was greater in comparison to those who did (OR = 2.51; 95%CI 1.26–5.04 and OR = 4.27; 95%CI 1.80–10.12). Physical abuse increased the odds of having marked/severe PD (OR = 2.28; 95%CI 1.26–4.12). Having experienced three or more mistreatment types increased the odds of at least moderate PD (OR = 2.90; 95%CI 1.30 - 35.74) and marked/severe PD (OR=3.86; 95%CI 1.58–9.42).

**Limitations:**

Disrespect and abuse experiences during childbirth were self-reported.

**Conclusions:**

Disrespect and abuse during childbirth increased the odds of PD three months after birth. Strategies to promote high quality and respectful maternal health care are needed to prevent mother-child adverse outcomes.

## Highlights

• 18% of the women experienced at least one mistreatment type during childbirth.
• Verbal abuse increased the likelihood of having postpartum depression.
• The effect of verbal abuse was greater among women without antenatal depression.
• Physical abuse increased the odds of having moderate/severe postpartum depression.

## 1 Introduction

Facility-based deliveries and skilled attendance at birth are key drivers of reductions in maternal morbidity and mortality globally (Joseph et al., 2016). Despite the substantial progress in the coverage of these key delivery indicators, inadequate access to comprehensive obstetric care remains an important challenge for many women, particularly in the context of low and middle-income countries (Jewkes and Penn-Kekana, 2015). Respectful maternity care, including the prevention and elimination of disrespect and abuse of women during facility-based childbirth is a critical component to addressing Sustainable Development Goals to improve maternal and newborn health globally (WHO 2016, World Health Organization 2015).

Evidence suggests that many women around the world face disrespectful, abusive or neglectful treatment during institutional childbirth, that puts their lives and well-being at risk (Amroussia et al., 2017, Bhattacharya and Sundari Ravindran, 2018, Bohren et al., 2017, Bohren et al., 2015, Mesenburg et al., 2018, Raj et al., 2017, Warren et al., 2017). Disrespect and abuse of women during the process of childbirth constitutes a human rights violation, particularly in a period when the women are more vulnerable (The United Nations General Assembly 1948, Nations, 1993).Women´s experiences of disrespect and abuse often results from the nature of patient-provider interactions in the context of obstetric care and can be expressed as verbal, physical or sexual abuse, stigma and discrimination, neglect, and failure to meet standards of care and attention – such as privacy and confidentiality breaches, limiting access to information and medical procedures conducted without consent (Bohren et al., 2015, Savage and Castro, 2017). They have also been linked to the institutional structures and processes that frame the practice of obstetric care in health systems and the persistence of structural gender inequalities in society (Betron et al., 2018, Sen et al., 2018a), being considered by some authors as a dimension of violence against women (Betron et al., 2018, Jewkes and Penn-Kekana, 2015).

In recent years, there have been important advances in documenting the burden of disrespect and abuse of women during maternity care (Savage and Castro, 2017). However, whilst the rapidly increase in the volume of research reporting prevalence and determinants in low and middle-income countries can contribute to policy (Savage and Castro, 2017), few studies have assessed the consequences of poor care during childbirth on the health of women and their newborns. Postpartum depression is common affecting women worldwide, with higher prevalence in low and middle-income countries (Fisher et al., 2012), and it has been associated with interpersonal difficulties, parenting problems and poorer child behavioral, attachment and cognitive outcomes (Jacques, 2019, Netsi et al., 2018, Stein et al., 2014). Although causes of perinatal depression are known to be multifactorial, negative birth experiences, such as feeling of abandonment during delivery, have been linked to the occurrence of psychiatric disorders, including depression and post-traumatic stress disorder in the postnatal period (De Schepper et al., 2016, Reed et al., 2017).

Using data from the 2015 Pelotas birth cohort, we investigated the association between disrespect and abuse of women during facility-based childbirth and maternal postpartum depression. A previous study in this cohort showed that 18% of the mothers experienced at least one type of disrespectful or abusive treatment during the process of childbirth (verbal abuse [10%], physical abuse [5%], denial of care [6%], undesired procedures [6%]), with a higher prevalence among women relying on the public health sector and those who delivered via cesarean section after going into labor (Mesenburg et al., 2018). Our study has two aims: (1) to examine the effect of the different types of disrespectful and abusive experiences (verbal abuse, denial of care, physical abuse, and undesired procedures) on maternal postpartum depression occurrence and, given that antenatal depression is a strong predictor of depression in the postpartum period and can influence how women perceive, internalize or justify experiences, (2) to explore if the associations differ according to women's antenatal depressive symptoms status.

## 2 Methods

### 2.1 Design and participants

The sample comprised participants from the 2015 Pelotas (Brazil) Birth Cohort Study, a population-based cohort of all live births from mothers living in the urban area of the city of Pelotas, Southern Brazil (Hallal et al., 2017). All women resident in the urban area of the city with confirmed pregnancy estimated delivery date in the year 2015 were invited to take part in the antenatal follow-up of the cohort. Eligible pregnant women were recruited from antenatal care health services and face-to-face interviews were conducted using structured questionnaires. Information on several maternal health pregnancy-related factors was assessed mid pregnancy (16–22 weeks of gestation) (Hallal et al., 2017). From January 1 to December 31, 2015, maternity hospitals were daily visited, and births detected. A total of 4333 women gave birth in the city's hospitals, of whom 4275 (98.7%) agreed to participate this study. Children were visited at home at 3 months, when 4110 face-to-face interviews were conducted (97.2%). Of these, 4087 interviews (95.6%) were done with the biological mothers. (Hallal et al., 2017) 73.8% of the mothers of live born children enrolled in the cohort were identified during pregnancy. Our final sample comprised data from biological mothers attending the antenatal and 3-month follow-ups of the cohort (*n* = 3065).

## 3 Measures

### 3.1 Disrespect and abuse of women during childbirth

Women´s disrespect and abuse experiences by health-care providers during facility-based childbirth were assessed during household interviews with the biological mothers three months after delivery. Self-reported information on verbal abuse, denial of care (abandonment of care), physical abuse and undesired procedures (non-consented care) during the process of childbirth were investigated using the following questions:

a) Physical abuse: “Has any professional ever pushed, hurt, beat, or held yourself strongly or conducted any examinations rudely or disrespectfully?”
b) Verbal abuse: “Has any professional been rude to you, cursed you or yelled at you, humiliated you or threatened not to assist you?”
c) Denial of care: “Has any professional refused to give you anything that you asked for, such as water or painkillers?”
d) Undesired obstetric procedures: “Has any professional ever conducted any procedure against your will, without explaining the need to conduct it, such as episiotomy or medication to induce labor?”

These questions were based on the questionnaire used in a large population-based survey carried on in Brazil which aimed to provide a panorama of labor and birth outcomes in the country (Leal et al., 2012).

For all forms of disrespect and abuse experiences, women were asked to consider the entire period they stayed in hospital, from arrival to discharge. Each question was coded as “yes” or “no”, and binary variables were created to indicate the occurrence of each disrespect and abuse type. Positive responses to each question were used to derive a score indicating the number of disrespect and abuse types experienced. An indicator variable was created whereby women were classified as having experienced 0, 1, 2 or 3/4 types.

### 3.2 Depressive symptoms

Symptoms of depression were assessed by face-to-face interviews using the Edinburgh Postnatal Depression Scale (EPDS) mid pregnancy (between the 16th and 24th week of gestation) and three months after delivery. The scale consists of ten items scored on a 4-point Likert scale (0–3) addressing common depressive symptoms experienced in the preceding week. A composite score is calculated by taking the sum of all items, ranging from 0 (absence of depressive symptoms) to 30 (highest score).(Cox et al., 1987) A positive screening for antenatal depression was defined as EPDS≥10, which is the recommended cut-off for screening in the Brazilian population (Santos et al., 2007). In the postpartum period, a cut-off point of ≥ 13 points in the scale was used to indicate the presence of at least moderate postpartum depression (primary outcome). This cut-off point has been shown to have a sensitivity of 59.6 (49.5–69.1) and specificity of 88.3 (83.9–91.9) for depression diagnosed by clinical interviews, taken as a gold standard in a previous study in this community (Santos et al., 2007). Additionally, we explored the threshold of 15 or more, which has been shown to have sensitivity of 40.4 (30.9–50.5) and specificity of 94.2 (90.7–96.6) to diagnose marked/severe cases of postpartum depression in the same community.

### 3.3 Confounding variables

Key confounding variables included: family income (measured in Brazilian reais and divided in quintiles), maternal education (up to 4, 5–8, 9–11, and 12 or more complete years of formal education), age (up to 19, 20–34, 35 or over), skin color (white/non-white), parity (0, 1 or more living children), cohabiting with a partner (yes/no), planned pregnancy (yes/no), fathers’ reaction when discovering the pregnancy (happy/other), previous history of depression (yes/no), pregnancy morbidities (self-reported gestational diabetes and hypertension), and delivery type (cesarean section or vaginal birth). Maternal characteristics were obtained from questionnaires administered in the antenatal and birth follow-ups.

### 3.4 Statistical analysis

The association between disrespect and abuse experiences during childbirth and postpartum depression occurrence was assessed using logistic regression analysis, computing odds ratios unadjusted and adjusted for key confounding variables. Second, we examined if the associations differ according to the woman´s antenatal depression status. We compared models with and without interaction terms, testing moderating effects of antenatal depressive symptoms using likelihood ratio tests. Finally, we assessed the effect of cumulative disrespect and abuse experiences during childbirth. Stata 13 software was used to conduct statistical analyses (StataCorp. 2013. Stata Statistical Software: Release 13. College Station, TX: StataCorp LP).

### 3.5 Ethics

This study was approved by the Research Ethics Committee of the School of Physical Education of the Federal University of Pelotas (CAAE 26746414.5.0000.5313) in February 5, 2014. All women provided written informed consent.

## 4 Results

Complete data were available for a total of 3065 women (biological mothers with complete information for both the main exposure and outcome, who participated in the antenatal component of the cohort study and had antenatal depressive symptoms assessed). Table 1 presents the sample characteristics and a comparison with the total cohort population. Most women had 9 or more years of formal education (69.8%), white skin color (72.8%), were living with a partner (87.2%) and delivered by c-section (67.1%). About half of women were aged 20 to 29 (47.8%) and were in their first pregnancy (51.9%). A history of depression was reported by 17% of the women and about 30% of women presented depressive symptoms during pregnancy (EPDS≥10). Any disrespect and abuse experience during childbirth was reported by 18% of the women, verbal and physical mistreatment by around 9% and 5%, respectively, and denial of care and undesirable procedures by 6% for both. A slightly lower proportion of women with low education, poorest (lower family income quintile), aged less than 19 years, having more than one child, and who delivered by c-section were observed in our sample, in comparison to the entire cohort sample.

**Table 1:** Sample description and comparison to the total cohort population, 2015 Pelotas (Brazil) Birth Cohort Study.

Characteristics	N sample	% (CI 95%)	N total cohort	% (CI 95%)
	3065		4275	
Maternal education (years)				
up to 4	212	7.2 (6.1–7.9)	391	9.2 (8.3–10.0)
5–8	709	21.7 (21.7–24.7)	1095	25.6 (24.3–27.0)
9 −11	1121	36.4 (34.9–38.3)	1458	34.1 (32.7–35.5)
12+	1022	33.4 (31.7–35.0)	1330	31.1 (29.7–32.5)
Family Income (quintiles)				
Poorest	526	17.2 (15.9–18.6)	846	19.8 (18.6–21.0)
2	617	20.1 (18.8–21.6)	859	20.1 (18.9–21.3)
3	625	20.4 (19.0–21.9)	853	20.0 (18.8–21.3)
4	636	20.8 (19.4–22.2)	856	20.0 (18.9–21.3)
Richest	659	21.5 (20.0–23.0)	859	20.1 (18.9–21.3)
Skin color				
White	2227	72.8 (71.2–74.3)	3024	70.9 (69.5–72.2)
Other	833	27.2 (25.7–28.8)	1244	29.2 (27.8–30.5)
Maternal age				
up to 19	399	13.0 (11.9–14.3)	622	14.6 (13.5–15.6)
20–29	1464	47.8 (46.0–49.5)	2017	47.2 (45.7–48.7)
30+	1201	39.2 (37.5–40.9)	1635	38.3 (36.8–39.7)
Multipara	1540	48.1 (46.3–49.8)	2137	50.0 (48.5–51.5)
Living with partner	2673	87.2 (86.0–88.4)	3667	85.8 (84.7–86.8)
Delivery type				
Vaginal	1008	32.9 (31.3–34.6)	1489	34.8 (33.4–36.3)
Cesarean section	2056	67.1 (65.4–68.7)	2785	65.2 (63.7–66.6)
History of depression	525	17.2 (15.9–18.5)	–	–
Antenatal depression^a^	901	29.4 (27.8–31.0)	–	–
Verbal abuse	270	8.8 (7.9 – 9.9)	378	9.2 (8.4 – 10.2)
Physical abuse	135	4.4 (3.7 – 5.2)	183	4.5 (3.9 – 5.2)
Denial of care	172	5.6 (4.9 – 6.5)	240	5.9 (5.2 – 6.6)
Undesirable/unconsented procedures	176	5.7 (4.9 – 6.6)	236	5.8 (5.1 – 6.5)
Any disrespect and abuse experience	552	18.0 (16.7 – 19.4)	749	18.3 (17.2 – 19.5)

a Assessed in the antenatal follow-up wave of the cohort using the Edinburgh Postnatal Depression Scale (the cut-off of 10 points was used to indicate a positive screening for antenatal depression).

Mean EPDS scores and the proportions of women who screened positive for at least moderate postpartum depression and marked/severe postpartum depression are shown in Table 2. In the sample studied mean EPDS score was 5.73 (SD 4.63) while the proportions of women with at least moderate postpartum depression (EPDS≥13) and marked to severe postpartum depression (EPDS≥15) were 9.4 and 5.7, respectively. These proportions were slightly higher in the total cohort population and significantly higher for among women who screened positive for antenatal depression.

**Table 2:** Mean EPDS scores and prevalence of postpartum depression, 2015 Pelotas (Brazil) Birth Cohort Study.

Outcome / Sample	All (*N* = 3065)	No antenatal depression (*N* = 2164)	Antenatal depression (*N* = 901)	Total cohort (*N* = 4275)
EPDS score, mean (SD)	5.73 (4.63)	4.35 (3.52)	9.06 (5.25)	6.00 (4.84)
EPDS score ≥13, N (%)	287 (9.4)	66 (3.1)	221 (24.5)	453 (11.1)
EPDS score ≥15, N (%)	174 (5.7)	30 (1.4)	144 (16.0)	278 (6.8)

Table 3 presents the unadjusted and adjusted odds ratios of having postpartum depression according to the occurrence of disrespect and abuse during childbirth. After controlling for potential confounders, women who experienced verbal abuse were 1.6 times more likely of having at least moderate postpartum depression (EPDS≥13) than those who did not (OR 1.58 95%CI 1.06–2.33). An interaction between antenatal depressive symptoms and disrespect and abuse of women during childbirth on postpartum depression occurrence was observed (*p* = 0.09). When the analyses were stratified by presence of antenatal depressive symptoms, verbal mistreatment during childbirth was associated with an increased likelihood of postpartum depression among women who did not experience antenatal depressive symptoms (OR 2.37 95%CI 1.14–4.91) but not among those who did (OR 1.16 95%CI 0.71–1.87).

**Table 3:** Unadjusted and adjusted odds ratio and 95% confidence interval of having postpartum depression according to the disrespect and abuse experiences during childbirth, 2015 Pelotas Birth Cohort Study.

Variable	All (*N* = 3065)				No antenatal depression (*N* = 2164)				Antenatal depression (*N* = 901)				Interaction term p value
	Unadjusted		Adjusted*		Unadjusted		Adjusted*		Unadjusted		Adjusted*		
*Postpartum depression (EPDS score ≥13)*													
Any disrespect and abuse	1.54	1.15–2.05	1.31	0.96–1.79	1.79	1.01–3.18	1.69	0.93–3.09	1.02	0.71–1.45	1.04	0.71–1.53	0.15
Verbal abuse	1.85	1.30–2.65	1.58	1.06–2.33	2.51	1.26–5.04	2.37	1.14–4.91	1.07	0.69–1.66	1.16	0.71–1.87	0.09
Denial of care	1.70	1.09–2.66	1.48	0.91–2.41	1.98	0.83–4.69	1.80	0.72–4.45	1.30	0.74–2.26	1.24	0.67–2.28	0.47
Physical abuse	1.63	0.99–2.69	1.54	0.90–2.65	1.20	0.37–3.91	1.22	0.36–4.06	1.40	0.76–2.57	1.58	0.81–3.07	0.74
Undesired procedures	1.41	0.89 2.25	1.34	0.82–2.20	1.62	0.64–4.12	1.71	0.65–4.50	0.99	0.56–1.74	1.05	0.58–1.93	0.48
*Postpartum depression (EPDS score ≥15)*													
Any disrespect and abuse	1.86	1.32–2.63	1.56	1.07–2.27	2.39	1.08–5.25	1.99	0.87–4.56	1.25	0.84–1.87	1.30	0.84–2.01	0.26
Verbal abuse	2.10	1.37–3.21	1.69	1.06–2.70	4.27	1.80–10.12	3.70	1.48–9.25	1.12	0.67–1.86	1.17	0.66–2.05	0.03
Denial of care	1.91	1.13–3.23	1.56	0.86–2.80	1.38	0.32–5.87	1.01	0.22–4.63	1.64	0.90–3.02	1.53	0.77–3.03	0.79
Physical abuse	2.36	1.37–4.07	2.28	1.26–4.12	2.85	0.85–9.60	2.42	0.68–8.67	1.83	0.95–3.52	2.08	1.01–4.28	0.61
Undesired procedures	1.35	0.75–2.43	1.32	0.71–2.46	2.19	0.65–7.34	2.25	0.64–7.95	0.87	0.43–1.74	0.98	0.47–2.03	0.28

⁎ Adjusted for maternal education, family income, skin color, age, parity, desire of pregnancy, marital status, father reaction when discovering pregnancy, pregnancy morbidities, deliver type and history of depression.

Considering the threshold for marked to severe depression (EPDS ≥ 15), adjusted associations were also significant for any type of disrespect and abuse experience and for physical abuse. Women reporting at least one type of disrespect and abuse were 1.6 times more likely to present postpartum depression (OR 1.56 95%CI 1.07–2.27) while those who experienced physical abuse were 2.3 times more likely than those who did not (OR 2.26 95%CI 1.26–4.08). In the stratified analyses, physical abuse during childbirth was associated with an increased odds of postpartum depression occurrence among women who experienced antenatal depressive symptoms (OR 2.08 95%CI 1.01–4.28) but not among those who did not (OR 2.42 95%CI 0.68–8.67), although no statistically significant interaction was found. The adjusted association between verbal abuse and the occurrence of marked to severe postpartum depression was also significant (OR 1.69 95%CI 1.06–2.70) as well as its interaction with antenatal depressive symptoms (*p* = 0.03). Stratified analyses showed similar patterns to those found for EPDS≥13. However, the odds ratio for marked to severe postpartum depression among women who did not experience antenatal depressive symptoms where even higher (OR 3.70 95%CI 1.48–9.25). There was no clear statistical evidence that denial of care and undesired obstetric procedures are associated with postpartum depression occurrence in the adjusted analyses for any of the EPDS thresholds used.

Table 4 shows the odds of having postpartum depression according to the cumulative number of disrespect and abuse types experienced during childbirth. In the total sample, a significantly positive association can be observed between the odds of postpartum depression and the number of disrespect and abuse experiences for both EPDS criterion. Women who experienced three or more types of disrespect and abuse were nearly 3 times and 4 times more likely to have postpartum depression than those who did not experience any form, using the threshold of ≥13 and ≥15, respectively. Among women who did not screen positive for antenatal depression the OR of having postpartum depression was about 7 (OR 6.87 95%CI 1.32–35.74) for those who reported three or more types of disrespect and abuse experiences.

**Table 4:** Unadjusted and adjusted odds ratios and 95% confidence intervals of having postpartum depression according to the cumulative number of disrespect and abuse types experienced during childbirth, 2015 Pelotas Birth Cohort Study.

Variable	All (*n* = 3065)				No antenatal depression				Antenatal depression			
	Unadjusted		Adjusted*		Unadjusted		Adjusted*		Unadjusted		Adjusted*	
*Postpartum depression (EPDS score ≥13)*												
	*<0.001*ꝉ		*0.009*ꝉ		*0.026*ꝉ		*0.043*ꝉ		*0.430*ꝉ		*0.306*ꝉ	
Any disrespect and abuse	1.29	0.92–1.83	1.11	0.77–1.60	1.62	0.83–3.15	1.49	0.74–2.98	0.87	0.57–1.33	0.88	0.56–1.38
2 types	2.04	1.19–3.50	1.60	0.88–2.92	1.98	0.60–6.54	2.02	0.59–6.97	1.27	0.67–2.49	1.26	0.61–2.58
3–4 types	2.72	1.29–5.73	2.90	1.30–6.48	3.24	0.74–14.14	3.26	0.70–15.10	1.66	0.65–4.24	2.23	0.81–6.14
*Postpartum depression (EPDS score ≥15)*												
	*<0.001*ꝉ		*<0.001*ꝉ		*0.009*ꝉ		*0.034*ꝉ		*0.226*ꝉ		*0.179*ꝉ	
Any disrespect and abuse	1.70	1.14–2.54	1.45	0.94–2.23	2.09	0.83–5.23	1.72	0.66–4.47	1.22	0.77–1.95	1.27	0.77–2.10
2 types	1.79	0.88–3.63	1.19	0.52–2.72	1.54	0.20–11.63	1.36	0.17–10.91	1.11	0.51–2.45	0.98	0.39–2.46
3–4 types	3.61	1.58–8.27	3.86	1.58–9.42	7.83	1.72–35.45	6.87	1.32–35.74	1.85	0.66–5.22	2.48	0.81–7.56

⁎ Adjusted for maternal education, family income, skin color, age, parity, desire of pregnancy, marital status, father reaction when discovering pregnancy, pregnancy morbidities, deliver type and history of depression.

ꝉ *p*-values for linear trend.

## 5 Discussion

We assessed the association between disrespect and abuse of women during childbirth and postpartum depression occurrence in a large prospective population-based cohort study. Our study showed increased odds of having postpartum depression among women who were exposed to verbal or physical abuse during childbirth. The stratified analyses by antenatal depressive symptoms showed a higher likelihood of both at least moderate and marked to severe postpartum depression among women who did not screen positive for antenatal depression and reported having experienced verbal abuse, and a greater effect of physical abuse on those previously presenting antenatal depressive symptoms. In addition, having experienced three or more disrespect and abuse types increased the likelihood of having postpartum depression exponentially, particularly among those who were not depressed during pregnancy. This suggests a multiplicative interaction between the different forms of disrespect and abuse experienced in increasing the risk of maternal postpartum depression occurrence.

Although the topic of respectful maternity care has gained increased attention in the recent years with several studies pointing out that disrespect and abuse during childbirth in health-facilities is frequent in different contexts and countries (Bohren et al., 2015), limited research has evaluated the consequences of such experiences on the health of mothers and children. Consequences of disrespect and abuse during childbirth on women's mental health outcomes have been mainly focused on the risk of post-traumatic stress disorder (Reed et al., 2017). To our knowledge only one previous study evaluated the influence of disrespect and abuse experiences during childbirth on postpartum depression occurrence (Souza et al., 2017). Findings of this study are consistent to ours as the authors found a positive cross-sectional association between institutional violence in obstetric care and postpartum depression, with physical violence between the parturient and health care providers as the most important determinant. In our study, however, both physical abuse and verbal abuse were associated with an increased risk of postpartum depression (slightly higher odds was found for physical abuse) and strengthen the evidence of other studies that identify the relationship between care providers and women as critical to the birth experience (Asefa et al., 2018, Peca and Sandberg, 2018, Reed et al., 2017).

Interestingly, in our study, distinct patterns of association were observed depending on the presence of maternal antenatal depressive symptoms. The effect of verbal abuse exposure on increasing the risk of postpartum depression was significantly higher for women who were not depressed during pregnancy. The lack of association between verbal abuse and postpartum depression among women who present symptoms of depression during pregnancy, however, need to be interpreted with caution. The literature suggests that many forms of disrespect and abuse during childbirth are normalized so they are not considered a problem; as a result, women have low expectations of care (Betron et al., 2018). Women who were depressed have specific personal characteristics that might influence the perceived care they received. In this cohort population, main predictors of antenatal depression have been previously related to a context of disadvantage (e.g. low maternal education, low family income, higher parity, not living with a partner, unplanned pregnancy). (Coll et al., 2017) It is possible, therefore, that women living in adversity lack knowledge of their rights and choices regarding obstetric care and that these social determinants of health have a greater impact on their risk of perinatal depression. Even so, perceptions of physical abuse, generally a more severe form of mistreatment, were related to an increased risk of postpartum depression among this subgroup of women.

The findings of the present study need to be interpreted considering certain limitations. Disrespect and abuse during childbirth was assessed according to women´s perceptions of their experiences and it remains unclear from the literature whether recall bias introduced by self-report measure would lead to under or over-reporting. The literature has been suggesting that recall may be more accurate in the postpartum period (as conducted in our study) than immediately following delivery when women are physically exhausted and have not had time to mentally process the events that occurred during childbirth (Sando et al., 2017). Also, although we report on four of the most common types of disrespect and abuse experiences during childbirth, further studies would benefit from addressing other important components of mistreatment during maternity care, such as stigma and discrimination based on specific patient characteristics, health systems conditions and constraints, and the general satisfaction and support received (Bohren et al., 2015, d'Orsi et al., 2014, Sando et al., 2017).It should also be noted that the number of disrespect and abuse episodes, the type of provider involved in mistreatment perpetration during childbirth and its severity were not evaluated in the present study. However, the experience of distinct types of disrespect and abuse by the same woman could be a proxy of severity. Finally, it is possible that depression status at the time of the interview could have influenced women's perceptions of disrespect and abuse during childbirth, leading to misreporting of experiences (Fergusson et al., 1993).

Another limitation is that the occurrence of maternal depression was assessed by a screening tool, not by clinical diagnosis. Although the EPDS was previously validated at community level in the same setting (Santos et al., 2007), the possibility of underreporting of depressive symptoms should be considered. Finally, the fact that we restricted our analyses to the women who were interviewed in the antenatal follow-up of the cohort could have introduced some bias in the effect estimates found. However, although this sample presents some differences in relation to the entire cohort, sensitive analyses showed similar association estimates among both populations (see Table S1). Reduced statistical power could also have been an issue, especially when we stratified our sample and restricted it to those who had information during pregnancy. Reassuringly, however, most of the associations found were consistent across the different analyses performed, and although the magnitude of odds ratios from the analysis conducted with the whole sample slightly changed because modification of depression during pregnancy, the association persisted.

Our study has several strengths. As far as we are aware, this is the first longitudinal study to evaluate the association between disrespect and abuse of women by health-care providers during childbirth and postpartum depression occurrence. In contrast to the only other study which has evaluated this association, our study included all women gave birth in all hospitals from Pelotas (about 99% of total births). Furthermore, we investigated how antenatal depressive symptoms interacts with disrespect and abuse exposure during childbirth and modifies the associations.

Given the potential negative consequences of postpartum depression, the crucial question is how to eliminate disrespectful and abusive practices during childbirth? Critical drivers of disrespect and abuse have been related to structural and systemic gender inequalities determined by social norms that affect both clients and providers (Betron et al., 2018, Sen et al., 2018b). Ideally, the change in these norms perpetuated for centuries would lead to a significant decrease in these behaviors that frame the practice of obstetric care. However, these are slow and gradual processes and, given the serious consequences of the problem to public health, implementation of response strategies is urgently needed.

Health-care providers should ensure high-quality and respectful care to women and their newborns during childbirth and labor, but evidence shows that some providers have misconceptions about what constitutes an acceptable behavior (WHO, 2016). Globally, the process of childbirth needs to be rethought and reformulated, so that women regain the leading role and control over their bodies. Although good obstetric practices are widely recognized (WHO, 2016), they are not generally incorporated into routine of health services. Currently, childbirth can be a “dehumanized” and excessively medicalized event, where the health professional determines the entire process, which favors the perpetration of this kind of gender-based violence (Jewkes and Penn-Kekana, 2015). This was observed in our data, where more than 65% of deliveries were Caesarian -section (Barros et al., 2019) and disrespect and abuse experiences were reported by 18% of the mothers (Mesenburg et al., 2018). Therefore, it is essential to reformulate the training of health professionals to promote a more humanized view to both parturient and patients in general. In the context of secondary prevention, strategies for the systematic diagnoses of postpartum depression and service support should be implemented to facilitate the early identification of incident cases, so that they receive adequate treatment and support.

Our findings emphasize the urgent need for strategies to promote high-quality and respectful maternal health care and prevent mother-child adverse outcomes related to postpartum depression. Health care providers require training and support, to enable them to practice in ways that optimize psychological outcomes for women. To understand the health care providers views on disrespect and abuse during childbirth would be of great value to guide the design of effective approaches to eliminate disrespectful and abusive practices during childbirth. In this context, special attention should be given to the potential incident cases of postpartum depression. Future studies need to focus both on the effects of disrespect and abuse in the health of mothers and their children, but also on assessment of strategies for prevention and control.

## Details of ethics approval

This study was approved by the Research Ethics Committee of the School of Physical Education of the Federal University of Pelotas (CAAE 26746414.5.0000.5313). All women provided written informed consent.

## Funding

This study is based on data from the 2015 Pelotas (Brazil) Birth Cohort Study which is currently supported by the Wellcome Trust (grant 095582/z/11/z), the Brazilian National Research Council (CNPq) and the Coordination for the Improvement of Higher Education Personnel (CAPES).

## CRediT authorship contribution statement

**Mariangela Freitas Silveira:** Conceptualization, Writing - original draft, Formal analysis, Writing - review & editing. **Marilia Arndt Mesenburg:** Conceptualization, Writing - original draft, Formal analysis, Writing - review & editing. **Andrea Damaso Bertoldi:** Writing - review & editing. **Christian Loret De Mola:** Formal analysis, Writing - review & editing. **Diego Garcia Bassani:** Writing - review & editing. **Marlos Rodrigues Domingues:** Writing - review & editing. **Alan Stein:** Writing - review & editing. **Carolina V N Coll:** Conceptualization, Writing - original draft, Formal analysis, Writing - review & editing.

## Declaration of Competing Interest

The authors declare no conflict of interest.

## Appendix Supplementary materials

Image, application 1

Image, application 2
