# Supplementary material for: The association between disrespect and abuse of women during childbirth and postpartum depression: Findings from the 2015 Pelotas birth cohort study
Source: J Affect Disord. 2019 Sep 1;256:441–7. doi: 10.1016/j.jad.2019.06.016 (PMC6880287; doi:10.1016/j.jad.2019.06.016)
Supplement: Supplementary file 2 [file mmc2.docx]

Table S1. Unadjusted and adjusted associations between mistreatment of women during childbirth experiences and postpartum depression in the total cohort population, 2015 Pelotas Birth Cohort Study.

| **Variable** | **All (n=4275)** | | | |
| --- | --- | --- | --- | --- |
|  | **Unadjusted** | | **Adjusted*** | |
| *Postpartum depression (EPDS score ≥13)* |  |  |  |  |
| Any mistreatment | 1.87 | 1.50 - 2.34 | 1.30 | 0.96 - 1.78 |
| Verbal abuse | 2.07 | 1.57 - 2.73 | 1.59 | 1.08 - 2.34 |
| Denial of care | 1.96 | 1.39 - 2.75 | 1.51 | 0.94 - 2.44 |
| Physical abuse | 1.98 | 1.35 - 2.91 | 1.49 | 0.87 - 2.54 |
| Undesired procedures | 1.70 | 1.20 - 2.44 | 1.32 | 0.81 - 2.17 |
| *Postpartum depression (EPDS score ≥15)* |  |  |  |  |
| Any mistreatment | 2.34 | 1.80 - 3.05 | 1.54 | 1.07 - 2.24 |
| Verbal abuse | 2.40 | 1.74 - 3.32 | 1.72 | 1.09 - 2.73 |
| Denial of care | 2.45 | 1.67 - 3.60 | 1.62 | 0.92 - 2.88 |
| Physical abuse | 2.53 | 1.65 - 3.88 | 2.17 | 1.21 - 3.91 |
| Undesired procedures | 1.77 | 1.16 - 2.72 | 1.27 | 0.68 - 2.37 |

* Adjusted for maternal education, family income, skin color, age, parity, desire of pregnancy, marital status, father reaction when discovering pregnancy, pregnancy morbidities, deliver type and history of depression.
